# Electrical and optical study of nerve impulse-evoked ATP-induced, P2X-receptor-mediated sympathetic neurotransmission at single smooth muscle cells in mouse isolated vas deferens

**DOI:** 10.1016/j.neuroscience.2007.05.044

**Published:** 2007-08-10

**Authors:** J.S. Young, K.L. Brain, T.C. Cunnane

**Affiliations:** Department of Pharmacology, University of Oxford, Mansfield Road, Oxford OX1 3QT, UK

**Keywords:** calcium imaging, confocal microscopy, discrete event, EJP, NCT, BAPTA, 1,2-bis(o-aminophenoxy)ethane-N,N,N′,N′-tetraacetic acid, DE, discrete event, EJP, excitatory junction potential, GPVD, guinea-pig vas deferens, MVD, mouse vas deferens, NCT, neuroeffector calcium transient, *n*_NCT_, number of neuroeffector calcium transients per stimulus per cell, PSS, physiological salt solution, sEJP, spontaneous excitatory junction potential, SMC, smooth muscle cell

## Abstract

Simultaneous electrophysiology and confocal microscopy were used to investigate purinergic neurotransmission at single smooth muscle cells (SMCs) in mouse isolated vas deferens, and to explore the relationship between two high-resolution P2X-receptor-mediated measures of per pulse ATP release: transient peaks in the first time derivative of the rising phase of excitatory junction potentials (EJPs) recorded in single SMCs (‘discrete events’; DEs) and neuroeffector Ca^2+^ transients (NCTs) in the impaled SMCs.

This study shows that discrete events represent neurotransmitter release onto the impaled cell. First, the median amplitude of the first derivative of the EJP was larger when there was a coincident NCT in the impaled cell, compared with instances when no coincident NCT occurred. Second, the time-to-peak amplitude of the first derivative was shorter if there was a coincident NCT in the impaled cell, compared with when no coincident NCT was observed within the field.

Surprisingly, first derivative amplitude increased with the distance (of the corresponding NCT) from the microelectrode. The microelectrode did not locally inhibit the functional quantal size as there was no effect of distance on the normalized NCT amplitude. When the significant effect of distance (between the microelectrode and NCTs) on the first derivative amplitude was removed, there was no correlation between the unstandardized residual (of distance vs. first derivative amplitude) and NCT amplitude.

The absence of a correlation between DE and NCT amplitudes suggests that the NCT amplitude is a poor measure of quantal size. The usefulness of NCTs hence lies primarily in locating neurotransmitter release and measuring changes in local release probability.

Packets of ATP, released from sympathetic nerves onto smooth muscle cells (SMCs), cause a local depolarization; the coordination of contraction is widely assumed to arise because the SMCs are electrically coupled (Brading, 1999). This may not apply to SMCs in the mouse vas deferens (MVD), however. There are several studies that suggest that these SMCs are only weakly electrically-coupled (Holman et al., 1977; Blakeley et al., 1989; Young et al., 2007); for example, the mean input resistance of surface SMCs (176±18 MΩ; Young et al., 2007) is comparable to that of dispersed cells (331±43 MΩ; Blakeley et al., 1989) and significantly greater than that recorded from syncytial smooth muscle (5–30 MΩ; Bennett, 1967; Holman et al., 1977; Manchanda and Venkateswarlu, 1999). The capacitance is also inconsistent with syncytial coupling. Surface cells of the MVD, with a membrane time constant of 8.6 ms (Blakeley et al., 1989; equal to RC), have a capacitance of 48 pF; a coupled network of about 100 arteriole SMCs (Hirst and Neild, 1980) has a capacitance of 5.9 nF [calculated from the initial *dV*/*dt* we measure as 42 mV/s during a 0.25 nA current injection in their Fig. 4 and noting that *C*=*I*/(*dV*/*dt*)]. The ratio of capacitances between these systems (1:100) suggests that the ratio of coupled cells is also 1:100, and hence that the surface cells in the vas deferens are electrically isolated.

This conclusion is supported by a recent comparison of α-latrotoxin (25 pM)-stimulated spontaneous excitatory junction potentials (sEJPs) and simultaneously recorded neuroeffector Ca^2+^ transients (NCTs, Brain et al., 2002, 2003) in the same SMCs in the MVD (Young et al., 2007). Coincident sEJPs and NCTs were variable, but positively correlated in amplitude. Such a correlation suggests that the left-skewed amplitude distribution of sEJPs is not due to electrotonic attenuation of effects of ATP packets released onto neighboring SMCs within the syncytium, as earlier assumed (Purves, 1976; Tomita, 1970). More likely is that every sEJP was triggered by release of a single packet of ATP directly onto the impaled SMC and that the amplitude variation was due either to variability in the size of the released ATP packets, or in the responses of P2X-receptors on the SMC surface (Young et al., 2007).

The question asked in the present study is if nerve impulse-evoked EJPs in MVD are also caused exclusively by direct impact of released ATP packets onto P2X-receptors of the impaled SMC, or are in part due to spread of the effects of ATP packets released onto nearby SMCs. Based on studies in guinea-pig vas deferens (GPVD) or MVD of both EJPs and their first time derivative, the ‘discrete events’ (DEs; Blakeley and Cunnane, 1978, 1979), the conclusion was drawn that the nerve impulse–evoked EJP in single SMCs has two components: (i) a non-intermittent, non-fluctuating ‘slow’ component, and occasionally, superimposed on that, (ii) a ‘fast’ component, fluctuating in amplitude. The slow component was thought to be a population response to asynchronous release of single ‘packets’ of ATP from 1% to 3% of the many varicosities innervating the SMC bundle, spreading to the impaled SMC, and the fast component the response to single packets of ATP released from one to three of the much fewer varicosities directly innervating the impaled SMC. Each nerve impulse evoked an EJP but often, especially in GPVD, failed to trigger a DE (Blakeley and Cunnane, 1978, 1979; Cunnane and Stjärne 1982, 1984), indicating that local action potential–evoked release of transmitter from individual sites was often highly ‘intermittent’ (*P* that a stimulus would give rise to a DE ranged from 0.02–0.5). In the MVD stimulus-locked DEs occurred in two separate latency bands, within each of which DEs were much less intermittent, *P* was relatively high and could approach unity (Blakeley and Cunnane, 1979). In both species, DEs within single latency bands were initially thought to represent release of 1 to 10 transmitter quanta per pulse from one or a few varicosities closely innervating the impaled SMC (Blakeley and Cunnane, 1979).

The validity of this view has later been questioned, as more rigorous criteria were set up to identify a DE as the image of the effect of release of transmitter from a single release site (Cunnane and Stjärne, 1984). Based on the occurrence of spontaneous and evoked DEs with closely similar amplitude and time-course (‘identical fingerprints’), the conclusion was drawn that per pulse transmitter release from the average individual varicosity (in both MVD and GPVD) is monoquantal and highly intermittent (*P*_DE_ 0.02–0.03 at 1 Hz stimulation; Cunnane and Stjärne, 1984). This high degree of intermittency of per pulse neurotransmitter release from single varicosities in MVD has recently been directly supported by study of NCTs in MVD (mean *P*_NCT_ 0.019 at 1 Hz stimulation, Brain et al., 2002).

In MVD the vast majority of axons are in close apposition (<50 nm separation) with adjacent SMCs in this tissue (Lane and Rhodin, 1964), which contrasts with the GPVD, in which the majority (80%) are not in close apposition with the SMC (Merrillees, 1968). The reason why the probability that nerve impulse–released ATP will encounter the impaled SMC is so much higher in MVD than in GPVD is probably that the MVD has a higher density of close contact junctions.

The aims of this study were to investigate the relationship between the NCT and DE, and to establish whether the DE is indeed a measure of local neurotransmitter release. It should be noted that the amplitude of the first derivative of the voltage (*V*) with respect to time (*t*) is proportional to the maximum net current (*I*), as long as the capacitance (*C*) does not change [as *V*=*Q*/*C*, *dV*/*dt*=(1/*C*) · (*dq*/*dt*)=(1/*C*) · *I*]. The advantage of comparing DEs and NCTs is that both, ideally, reflect effects of opening the same P2X-receptors on the same SMC by the same released ATP packet. The difference is that DEs reflect the net flux of ions (including its small Ca^2+^ component) and NCTs the transient local increase in the Ca^2+^ concentration in the SMC resulting both from Ca^2+^ influx and its downstream effect: Ca^2+^-induced Ca^2+^ release from stores within the SMC (Brain et al., 2003). By combining confocal imaging with simultaneous intracellular electrophysiological recording in the mouse isolated vas deferens, it is possible to utilize the spatial information of NCTs coincident with EJPs to determine whether DEs are indeed indicative of local neurotransmitter release.

## Experimental procedures

### Ca^2+^ indicator loading

Eight- to 12-week-old Balb/c mice (Harlan, Bicester, Oxfordshire, UK) were killed by cervical fracture and both vasa deferentia removed. Efforts were made to minimize the number of animals used and their suffering; all experiments were carried out in accordance with the UK Animals (Scientific Procedures) Act 1986 and European Communities Council Directive 86/09/EEC. The connective tissue around each vas deferens was carefully dissected in order to obtain clear images of SMCs and to remove any ganglia located close to the prostatic end.

Each vas deferens was then exposed to 10 μM Oregon Green 488 1,2-bis(o-aminophenoxy)ethane-N,N,N′,N′-tetraacetic acid (BAPTA)-1 AM (Invitrogen, Paisley, Renfrewshire, UK) in 1% dimethyl sulfoxide/0.2% pluronic F-127 (Sigma-Aldrich, St. Louis, MO, USA) in physiological salt solution (PSS) for 2 h at 36 °C. Each tissue was then cut longitudinally to create a flat sheet and rinsed in PSS, bubbled with 95% O_2_/5% CO_2_, for at least 10 min. Tissues were pinned flat, serosal side up in a Sylgard-lined organ bath, and mounted on the stage of an upright confocal microscope. The PSS contained (mM): NaCl 118.4, NaHCO_3_ 25.0, NaH_2_PO_4_ 1.13, KCl 4.7, CaCl_2_ 1.8, MgCl_2_ 1.3 and glucose 11.1. The pH was maintained at 7.4 and the solution oxygenated by continuous bubbling with 95% O_2_/5% CO_2_.

### Confocal microscopy

The vas deferens was placed in a chamber that was continuously superfused with PSS (bath temperature 33–34 °C). Images were acquired using a Leica SP2 upright confocal microscope (Leica Microsystems, Milton Keynes, Buckinghamshire, UK). A series of 200 frames were captured at approximately 13.5 Hz to generate one image set. Such sets were acquired once every minute. Between 4 and 12 such sets were acquired for each SMC.

### Image analysis

Image analysis was performed using custom-written plug-ins for Image J (http://rsb.info.nih.gov/ij/download.html) to detect focal increases in fluorescence of the Ca^2+^ indicator. Data were exported to Excel (Microsoft, Redmond, WA, USA) for formatting and then to Spike 2 (Cambridge Electronic Design, Cambridge, Cambridgeshire, UK) for analysis in conjunction with electrophysiological recordings.

### Electrophysiology

Conventional intracellular recording techniques were used to record EJPs in SMCs (see Brock and Cunnane, 1992). Each vas deferens was superfused with PSS. Microelectrodes (tip resistances of 140–160 MΩ) were filled with a potassium salt of Oregon Green BAPTA-1 (100 μM filtered in 5 M potassium acetate; weight 10 kDa; Invitrogen). The membrane potential was measured with an Axoclamp 2B (Axon Instruments, Sunnyvale, CA, USA) in bridge mode.

Recordings of electrically-evoked EJPs were achieved by applying rectangular pulses (0.6 ms duration; voltage amplitude at twice the threshold for eliciting EJPs, typically around 15 V) at the onset of every tenth confocal acquisition (i.e. at 1.5 Hz) through Ag/AgCl electrodes positioned around the prostatic end of the vas deferens. A frame-coupled TTL output from the microscope enabled temporal correlation of electrophysiological and confocal recordings.

The voltage was digitized at 5 kHz with a PowerLab system (ADInstruments, Chalgrove, UK). Recordings of EJPs were exported from Chart 4.2 (ADInstruments) for analysis with Spike 2 (Cambridge Electronic Design). The amplitudes of EJPs were calculated using Chart 4.2 (ADInstruments). The first derivative of the EJP was calculated using Derivative function of Chart 4.2 (ADInstruments); absolute amplitudes of peaks in the first derivative (DEs) were measured using a custom-written macro.

The microelectrode tip (or part of it) was always within the field of view of the confocal image.

### Analysis of correlations between NCTs and EJPs

The peak amplitudes and timings of both EJPs and NCTs were calculated using a custom-written script for Spike 2. The threshold for determining NCTs varied between recordings from different SMCs. The threshold was adjusted until it provided a specificity comparable to manual counting (ΔF/F range of 0.03–0.05).

To investigate the temporal correlation between changes in the fluorescent signal and EJPs, the time from each change in fluorescent signal to the temporally nearest EJP was measured (referred to as delay). To determine whether these events displayed a significant temporal correlation, the distribution of delays was compared (using the Kolmogorov-Smirnov test; http://www.physics.csbsju.edu/stats/KS-test.n.plot_form.html) with the expected distribution under the assumption that EJPs and NCTs were uncorrelated. The expected distribution of delays is the double exponential or Laplace distribution (see Young et al., 2007).

Occasionally, >one coincident NCT was observed within the impaled cell. When >one coincident NCT was identified, the corresponding EJPs or DEs were not included in the analysis.

### Statistical analysis

For other statistical tests, the normality and homogeneity of variance were tested prior to statistical analysis using Kolmogorov-Smirnov and Levene’s tests, respectively (SPSS version 11, SPSS Inc., Chicago, IL, USA). The term *n*, used in the presentation of statistical analyses throughout, refers to the number of animals.

## Results

**Fig. 1:**
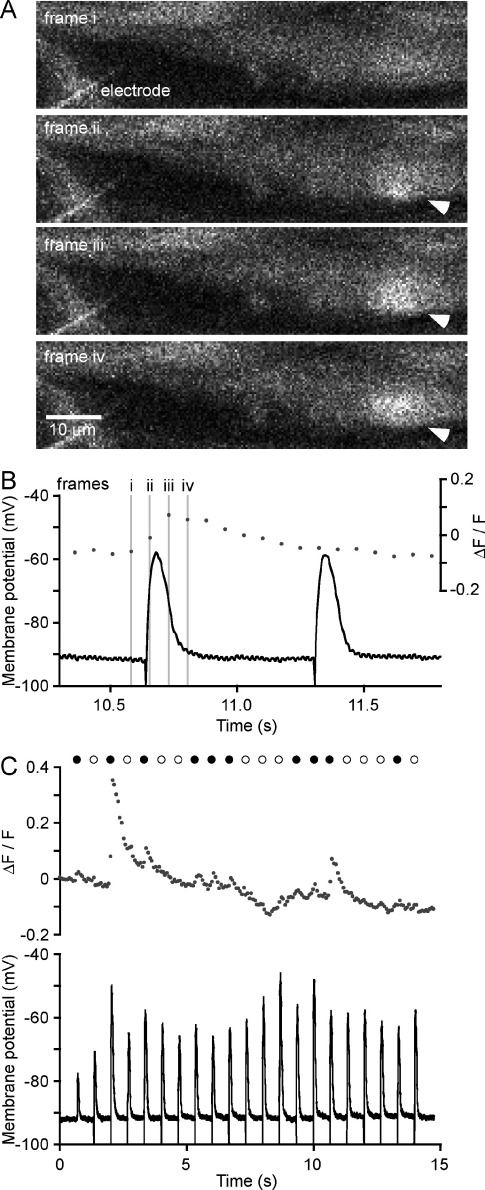
Electrical stimulation evokes EJPs and coincident NCTs in mouse isolated vas deferens smooth muscle. (A) A region of a SMC in a mouse isolated vas deferens (loaded with the Ca^2+^ indicator Oregon Green 488 BAPTA-1 AM) during an intracellular recording. Images, acquired at 13.5 Hz, are 4 consecutive frames of a 200 frame series. Electrical stimulation, occurring on frame ii, produced a NCT in a SMC (arrowhead). (B) Intracellular recording of a period that includes the same four frames that compose A, showing simultaneous recordings of membrane potential (black line) and whole cell fluorescence (gray dots). Note that there is no detectable increase in whole cell fluorescence coincident with the second EJP. (C) Simultaneous Ca^2+^ imaging and electrophysiology during a longer recording. Electrical stimulation pulses are indicated by circles; occasions where a NCT in the impaled cell was coincident with the EJP are denoted by filled circles, whereas occasions where there was no coincident NCT in that cell are denoted by open circles. The detection threshold for NCTs was a ΔF/F of 0.03. Stimulus artifacts were truncated at −100 mV (B, C).

**Fig. 2:**
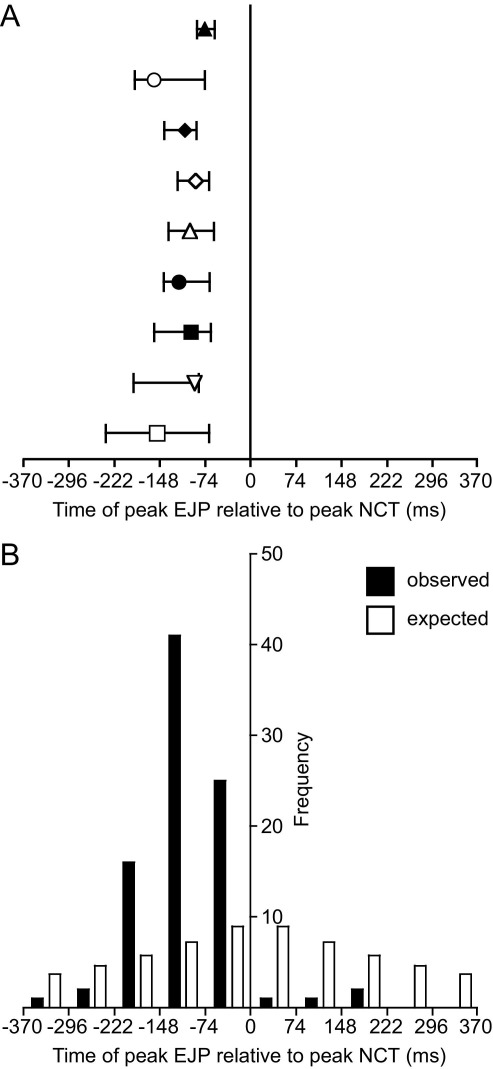
The temporal relationship between EJPs and coincident NCTs. (A) The median latency between the peak EJP and the peak NCT (*n*=9). Symbols denote different preparations. Error bars are the corresponding quartiles. (B) Data are presented for a typical experiment (filled bars) and expected values (open bars), as modeled by the Laplace distribution. The boundaries between intervals are shown at one frame width (74 ms).

Electrical stimulation (1.5 Hz, 15 V) evoked intermittent, focal increases in intracellular Ca^2+^ concentration within the SMC (NCTs; Fig. 1) and non-intermittent EJPs (Fig. 1B, C). The time from the peak of each NCT to the nearest EJP was measured in order to characterize the temporal relationship between these two measures of neurotransmission. The median time between the peak of an NCT and the peak of the nearest EJP ranged from −74 to −152 ms (*n*=9; Fig. 2A; i.e. with the NCTs peaking later). The frequency distribution of the temporal relationships between EJPs and NCTs was significantly different from a random (i.e. Laplace) distribution (Kolmogorov-Smirnov, *P*<0.001 in *n*=9 experiments; an example is shown in Fig. 2B).

Both methods of measuring neurotransmission used in this study-electrophysiology and monitoring NCTs with confocal microscopy-revealed large differences among the responses of individual surface SMCs. The resting membrane potentials of surface SMCs varied, ranging from −66.5 to −91.8 mV (−74.8±2.8 mV, mean±S.E.M., *n*=9). Likewise, the median EJP amplitude differed greatly between cells, ranging from 3.9–35.5 mV (*n*=9).

Although EJPs occurred with every electrical stimulus (i.e. *P*_EJP_=1), the probability that an electrical stimulus pulse would produce an NCT within the viewed area of the impaled cell, *P*_NCT_, was highly variable, ranging from 0.22–0.87. *P*_NCT_ was corrected for differences in the size of the region-of-interest (length 78–169 μm, *n*=9) with respect to the average SMC length (204 μm; Young et al., 2007) to give an estimate of the number of neuroeffector calcium transients per stimulus per cell (*n*_NCT_). The resulting estimated *n*_NCT_ varied between 0.39–1.76.

### Effects of local neurotransmitter release

**Fig. 3:**
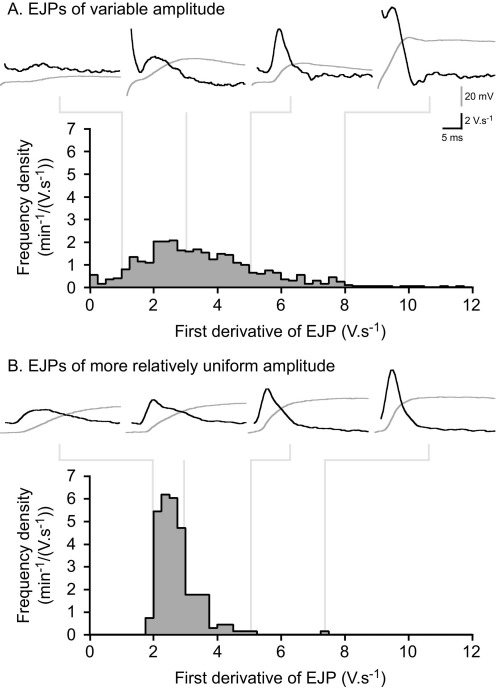
The amplitude distributions of the first derivatives of EJPs from two recordings; (A) from a cell in which EJPs were of variable amplitude (median: 17.2, upper quartile: 25.3, lower quartile: 10.2 mV) (614 EJPs), (B) from a cell in which EJPs were of a relatively more uniform amplitude (median: 28.6, upper quartile: 30.6, lower quartile: 27.2 mV) (205 EJPs). A sample of EJPs (gray lines) and their first derivatives (black lines) is shown above each first derivative amplitude frequency plot. Note the presence of a stimulus artifact (trace 2, A) and a first derivative with a short latency merging with its stimulus artifact (trace 4, A). Resting membrane potentials were −65 and −85 mV in (A) and (B), respectively.

In the present study, amplitudes of first derivatives of EJPs often occurred with an almost Gaussian distribution (Fig. 3). There were only two preparations (of nine) in which the amplitude-frequency distributions were highly left-skewed, which suggests that intermittency of high amplitude events in the first derivative is not measurable in most cells.

It was possible to classify a first derivative as a DE if there was a determinable peak, as adjudged by eye. In one recording (of nine), EJPs occurred which when differentiated revealed no DE. By contrast, there was one recording (of nine) in which a DE was elicited with almost every stimulation (149 DEs from 150 stimuli). To summarize: the probability that electrical stimulation would produce a DE, *P*_DE_, varied greatly between cells, ranging from 0 to 0.99.

Amplitudes of first derivatives of EJPs from each preparation varied greatly (e.g. Fig. 3A) and the variance in amplitudes of the first derivative also differed between experiments (e.g. compare Fig. 3A and Fig. 3B).

**Fig. 4:**
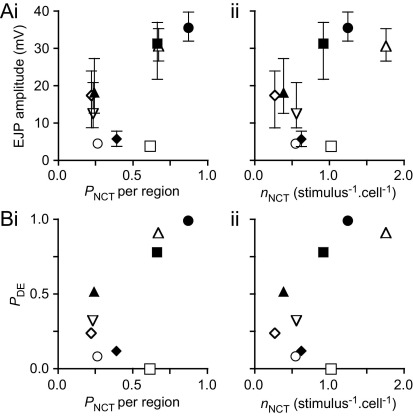
SMCs in which there is a high probability of a NCT occurring on electrical stimulus have a correspondingly higher median EJP amplitude (A) and probability of a DE occurring (B). (Ai) There was a positive correlation between the probability of an NCT occurring within the monitored region of the impaled cell (*P*_NCT_ per region) and the amplitude of EJPs recorded simultaneously from the same cell (Pearson one-tailed correlation, Pearson *r*=0.64, *P*<0.05). (Aii) There was no correlation between the *P*_NCT_ corrected for the size of the region-of-interest with respect to the mean SMC length (204 μm; Young et al., 2007) (estimated *n*_NCT_ per stimulus) and median EJP amplitude (Pearson *r*=0.31, *P*=NS). EJP amplitudes are median±quartiles. (Bi) There was a positive correlation between *P*_NCT_ per region and the probability of a DE occurring (*P*_DE_) on stimulation (Pearson *r*=0.43, *P*<0.05). (Bii) There was also a correlation between estimated *n*_NCT_ per stimulus and *P*_DE_ (Pearson *r*=0.33, *P*=0.05). *P*_NCT_ was corrected for the size of the region-of-interest with respect to the mean SMC length of MVD SMCs (204 μm; Young et al., 2007) to give an estimate of the number of NCTs per stimulus per cell (*n*_NCT_):$n_{\text{NCT}}({\text{stimulus}}^{-1}\cdot {\text{cell}}^{-1})=P_{\text{NCT}}\times \left(\frac{\text{mean}\;\text{SMC}\;\text{length}}{\text{length}\;\text{of}\;\text{SMC}\;\text{within}\;\text{field-of-view}}\right)$ This slightly underestimates the true number of NCTs per stimulus because it does not recognize the very occasional occurrence of more than one NCT in the field-of-view. Symbols correspond to those used in Fig. 2. Data from *n*=9 preparations.

The relationship between the probabilities that NCTs and coincident DEs occur on electrical stimulation was explored in order to understand whether DEs are indeed a measure of local neurotransmitter release, as NCTs indicate the location of the postjunctional P2X-receptors activated by the release of ATP. There was a positive correlation between *P*_NCT_ and both the median EJP amplitude (Fig. 4Ai) and *P*_DE_ (Fig. 4Bi). This suggests that all three techniques measure a common variable (neurotransmitter release). When *P*_NCT_ was corrected for differences in the size of the region-of-interest with respect to the average SMC length, to give an estimate of the *n*_NCT_, there was no correlation with median EJP amplitude (Fig. 4Aii), but there was a significant correlation with *P*_DE_ (Fig. 4Bii).

### Correlating events occurring at identified sites

**Fig. 5:**
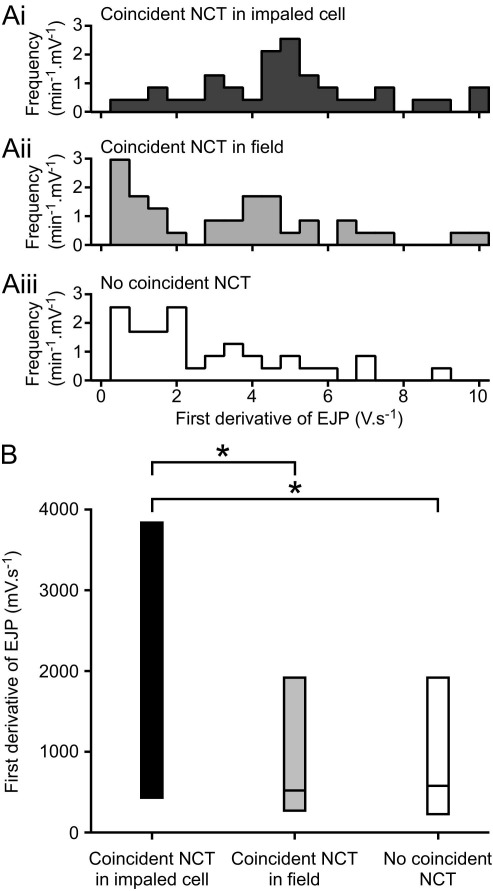
The presence of an NCT within the monitored region of the impaled cell coincides with increased amplitude in the first derivative of the EJP. (A) A typical example from one recording, showing that the amplitude distribution of the first derivative is shifted when an NCT was seen to occur within the impaled cell (i), compared with when an NCT occurred within the field, but not in the impaled cell (ii), or when no NCT is detected within the field (iii). (B) The amplitude of the median first derivative is significantly greater when there is a coincident NCT in the impaled cell than when an NCT occurs within the field but in another SMC (Wilcoxon signed rank test, *W*=30, *P*<0.05) or when no NCT is detected within the field (Wilcoxon signed rank test, *W*=39, *P*<0.05). Data are median and quartiles (*n*=9).

The hypothesis that a DE indicates local neurotransmitter release could be tested using positional information of identified NCTs. This allows coincident EJPs to be grouped according to their location within the field. The amplitude of the first derivatives of EJPs was of greater amplitude when an NCT was seen to occur in the impaled cell (i), compared with when an NCT occurred within the field but not in the impaled cell (ii), or when no NCT was detected within the field (iii) (Fig. 5).

**Fig. 6:**
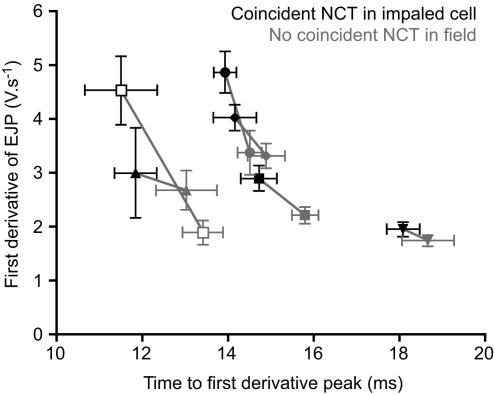
The occurrence of a NCT within the impaled cell (black) is coincident with an increased amplitude and a briefer time-to-peak of DEs, than when no coincident NCT is recorded within the field (gray). Data (mean±S.E.M.) are shown for the six of nine preparations in which there was a sufficient number of EJPs with a recognizable peak in the first derivative of the EJP to perform this analysis. Symbols denote different preparations.

The presence of an NCT in the impaled cell was not only correlated with the amplitude of the first derivative of a coincident EJP, but also its time-to-peak. The time-to-peak amplitude of the first derivative was briefer if there was a coincident NCT in the impaled cell, compared with when no coincident NCT was observed within the field (two-tailed paired *t*-test, *t*=4.9, *df*=5, *P*<0.01; Fig. 6). These observations are summarized in Fig. 6 for the six (of nine) preparations in which there were sufficient DEs to perform this analysis.

**Fig. 7:**
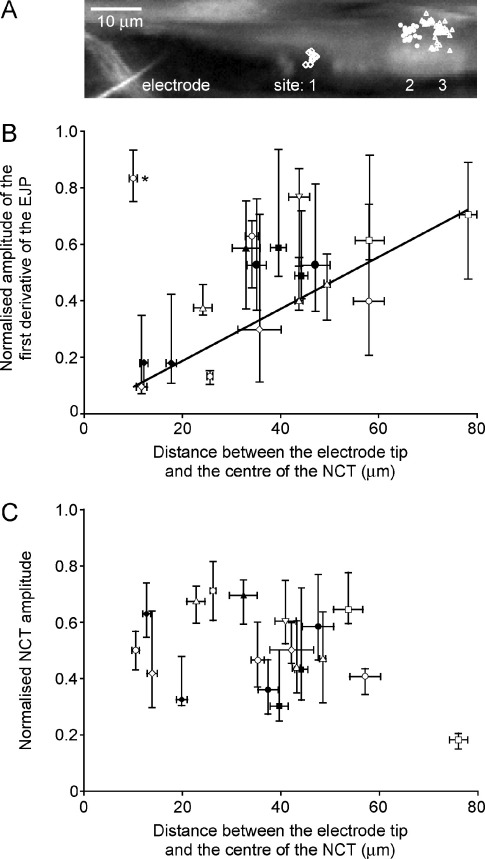
The amplitude of the first derivative of the EJP increases with the distance between recording electrode and the coincident NCT (i.e. the site of ATP binding). (A) DEs were grouped according to the site of coincident NCTs (*n*=1). A region of a SMC loaded with the Ca^2+^ indicator Oregon Green 488 BAPTA-1 AM during an intracellular recording. (B) There is a positive correlation between distance and normalized amplitude of the first derivative of the EJP (linear regression, *F*=16.1, *r*^2^=0.51, *P*<0.001, number of sites, *n*_*s*_=16, *n*=9). One outlier (asterisk) from this distribution was removed prior to fitting the linear regression. (C) There is no correlation between distance and normalized NCT amplitude (linear regression, *F*=2.7, *r*^2^=0.14, *P*=NS, *n*_*s*_=17). Sites were identified manually, by overlaying the coordinates of NCTs (exported from ImageJ) on an image of the SMC (Excel, Microsoft). The number of distinct sites of NCT occurrence varied (one to four) between preparations. Identical symbols represent data from the same preparation but from different sites, and the symbols used correspond to those used in Fig. 2. Distance from recording site is the mean±S.E.M. The first derivative, (Aii), normalized first derivative (B) and normalized NCT amplitude (C) are median and quartiles.

The positional information of NCTs (occurring on electrical stimulation within the impaled cell) was further used to determine if the distance between the microelectrode and the NCT affected the amplitude of the first derivative of the coincident EJP recorded from the (same) cell. NCTs often (*n*=7 of 9) occurred at >one (manually identified) site along the SMC (Fig. 7A), consistent with multiple sites of neurotransmitter action on each SMC. Surprisingly, the amplitude of the first derivative increased with the distance from the microelectrode (Fig. 7B). One possible explanation for this finding was that the presence of the microelectrode locally inhibited neurotransmitter release (or more specifically, that it inhibited the functional quantal size). There was, however, no effect of distance on the normalized NCT amplitude (Fig. 7C).

**Fig. 8:**
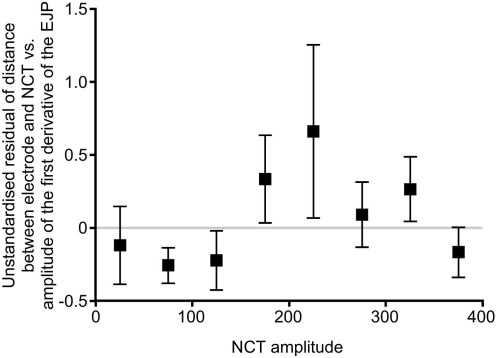
There is no correlation between the NCT amplitude and the unstandardized residual of the distance between the electrode and the NCT vs. the amplitude of the first derivative of the EJP. As the amplitude of the first derivative of the EJP varies with distance (Fig. 7B) whereas NCT amplitude does not, it is not possible to directly examine the correlation between the first derivative and NCT amplitudes. To account for the confounding effect of distance, a linear regression of the first derivative amplitude and distance was calculated (*F*=16.1, *r*^2^=0.51, *P*<0.001). The distance between the raw first derivative amplitude and the linear regression (unstandardized residual) was calculated (SPSS version 11, SPSS Inc.). There was no correlation between the unstandardized residual (of distance vs. amplitude of the first derivative) and the NCT amplitude. Data (*n*=9) were binned for presentation. The unstandardized residual is the mean±S.E.M.

The strong, positive effect of distance (between the microelectrode and the NCT) on the first derivative of the EJP was first removed before correlating the amplitude of the first derivative of the EJP and the amplitude of the coincident NCT. There was no correlation between the unstandardized residual (of distance vs. amplitude of the first derivative) and the NCT amplitude (Spearman two-tailed rank correlation, *r*=0.33, *n*=9, *P*=NS) (Fig. 8) (data shown for individual data sets in Supplementary Fig. 1).

### Variability in the latencies of DEs

DEs occurred at variable latencies with respect to the stimulus (e.g. Supplementary Fig. 2A). It was possible that this variable latency of DEs is attributable to the distance between the site of neurotransmitter release and the recording electrode. However, there was no correlation between distance (between recording site and the coincident NCT) and the latency of the DE for most (*n*=5 of 6) of the preparations in which there were enough observations to apply this test (Supplementary Fig. 2B). In one preparation (of six), however, there was a weak negative correlation between distance and latency; this is likely to have occurred by chance.

## Discussion

The primary aim of this study was to address the relationship between nerve impulse-evoked EJPs and peaks in their first time derivatives, DEs, and NCTs using a recently established simultaneous laser-scanning confocal microscopy and electrophysiological recording technique (Young et al., 2007). A second aim was to use the spatial information of NCTs coincident with DEs, to explore whether DEs represent the local release of ATP as a sympathetic neurotransmitter.

### Intermittent neurotransmitter release

That the electrically-evoked release of sympathetic neurotransmitter from individual varicosities may be highly intermittent was first suggested based on studies of the overflow of noradrenaline (Folkow et al., 1967). It has been more directly demonstrated by electrophysiological and optical analysis of the release of packets of ATP which trigger DEs (Blakeley and Cunnane, 1978), excitatory junction currents (Brock and Cunnane, 1987; Åstrand et al., 1988) and NCTs (Brain et al., 2002).

The large variation in the probability of neurotransmitter release, quantified as *P*_DE_, shown in the present study (*P*_DE_ ranged from 0–0.99) is consistent with the findings of previous studies reporting local intermittence measuring excitatory junctional currents (0.002–0.03, Cunnane and Stjärne, 1984) and a variable number of functional release sites on each SMCs. The cause of the variation at the level of the single release site is not presently known. First, the exogenous Ca^2+^ concentration strongly influences *P*_DE_, which increased from 0.34±0.04 at 1.1 mM Ca^2+^ to 0.53±0.03 at 2.1 mM (Blakeley et al., 1986). Second, the stimulus frequency is positively correlated with *P*_DE_. Stimulation at 0.5 Hz evoked 20 DEs per 500 stimuli, which increased to 60 per 500 at 1 Hz, and 150 per 500 at 2 Hz (Cunnane and Stjärne, 1984). Third, it is unclear how previous studies have distinguished a DE from more slow-rising changes in the first derivative. It may be more reasonable to classify events by eye based on their shape, rather than trying to determine a threshold of first derivative amplitude (e.g. Fig. 3). Cunnane and Stjärne (1984) were able to find an SMC in which *P*_DE_ was unusually low (0.002), although the density of such low probability recording sites remains to be established. It should be noted that a large variation in *P*_DE_ has been observed between MVD and GPVD. This difference may be attributed to differences in the density of close contact junctions between the two species (Lane and Rhodin, 1964; Merrillees, 1968).

The two methods of monitoring the postjunctional effects of ATP binding to P2X-receptors, DEs and NCTs, yield similar estimates of the probability of per pulse release from individual varicosities in the MVD. Study of the occurrence of spontaneous and evoked DEs with identical fingerprints gives a *P*_DE_ per single release site (i.e. varicosity) of 0.01–0.03 (Cunnane and Stjärne 1982, 1984) in the GPVD; study of the occurrence of NCTs triggered from single visualized varicosities at single junctions gives a *P*_NCT_ per varicosity of 0.019, range 0.001–0.1 (Brain et al., 2002).

Clearly, the optical method has the advantage over DEs that NCTs reveal activity in visually identified varicosities, whereas estimates based on DEs reflect, at best, activity in single release sites, assumed to be a single varicosity. But DEs do have one advantage over NCTs: reproducibility of the response to a released ATP packet. The close similarity in amplitude and time course (‘identity’) of repeated spontaneous and/or evoked fingerprinted DEs implies constancy, either of the size of ATP packets, or of the P2X-receptor-mediated response to a packet of ATP released from a particular site (Cunnane and Stjärne, 1984). This is in contrast to the variability of the, also P2X-receptor-mediated, NCTs responses to release of ATP from single identified varicosities, whose amplitude at a given junction can vary by more than ninefold (Brain et al., 2002). This is surprising, as both monitors of neurotransmission reflect effects of ATP-induced opening of the pore of the same P2X receptors. Apparently the answer is that DEs reflect the net flux of ions (including Ca^2+^) through this pore, but NCTs both Ca^2+^ influx and a significant but variable downstream effect: amplification by Ca^2+^-induced Ca^2+^ release (Brain et al., 2003).

### Estimates of innervation based on measures of intermittency

Using the calculated *n*_NCT_ (stimulus^−1^·cell^−1^; median 0.63, range 0.27–1.77, *n*=9) and *P*_NCT_ per varicosity (0.019; Brain et al., 2002) it is possible to estimate the number of varicosities that functionally innervate a single SMC to be 33 (median; range 14–93, *n*=9). These values may, however, underestimate the (presently not known) innervation density of MVD, due to the presence of varicosities in which the probability of neurotransmitter release is especially low (Brain et al., 2002).

### DEs are a measure of neurotransmitter release onto the impaled cell

This study shows for the first time that DEs represent neurotransmitter release onto the impaled cell. First, the median amplitude of the first derivative of the EJP was larger when an NCT occurred in the impaled cell, compared with when the NCT occurred in an adjacent cell or when there was no discernable NCT (Fig. 5). Second, the time-to-peak amplitude of the first derivative was briefer if there was a coincident NCT in the impaled cell, compared with when no coincident NCT was observed within the field (Fig. 6).

As the entire SMC could not be simultaneously viewed within one confocal optical section (mean field 126.5±9.8 μm), it is possible that an NCT could occur at a considerable distance from the recording electrode, i.e. in an area of the impaled SMC not viewed. Thus the spatial information associated with NCTs was used to test further the hypothesis that DEs are indicative of local neurotransmitter release. NCTs were observed to occur in distinct spatially-separate groups (Fig. 7Ai), consistent with previous observations and the finding that they occur close to varicosities (Brain et al., 2002), and so EJPs were classed according to the position of their coincident NCT on the SMC.

The absence of an effect of distance on (normalized) NCT amplitude (Fig. 7C) implies that neurotransmitter release is not affected by the location of the electrode. The amplitude of the first derivative of the EJP was, counterintuitively, positively correlated with the distance between the recording site and the position of NCT occurrence (Fig. 7B), suggesting that the electrode reduces the local current (see below). The absence of a fall in amplitude away from the electrode (Fig. 7B) argues against electrotonic decay; an absence of electrotonic decay implies that the cell will be isopotential.

The EJP amplitude in MVD is monotonically related to the net current through P2X_1_ receptors, while NCTs are initiated by local Ca^2+^ influx (Brain et al., 2002). Usually, the current through P2X_1_ receptors is dominated by Na^+^, with a small contribution by Ca^2+^; despite the observation that K^+^ is just as permeable as Na^+^ through P2X_1_ (Evans et al., 1996), its current through this receptor is negligible because the Nernst potential for K^+^ is close to the very negative resting membrane potential of the SMCs studied. One hypothesis is that a lower first derivative amplitude close to the electrode is caused by solute exchange through the electrode tip. As dyes, used to determine the position of the electrode, are seen to leak from the tip into the SMC (Young, unpublished observations), it is feasible that K^+^ (present at 5 M, with acetate) may also leak into the impaled cell. The resulting high local K^+^ concentration around the recording site would cause a K^+^ efflux through ‘open’ P2X_1_ receptors, reducing the amplitude of depolarizations close to the recording site. However, quantitative modeling of diffusion from the electrode tip with K^+^ leak out of the cell (see supplementary material), predicts that in the steady state, K^+^ will fall off exponentially with distance from the electrode with a space constant of around 585 μm. Hence, based on the best available parameters, this hypothesis is quantitatively inconsistent with the observed spatial zone of inhibition (Fig. 7B). We have not identified an alternative well-supported hypothesis, although it is possible that some unknown factor may locally inhibit Na^+^ but not Ca^2+^ conductance through the P2X_1_ receptor.

### The differing latencies of DEs are not due to distance between release and recording site

DEs occurred with differing latencies (e.g. Supplementary Fig. 2A) as previously described (Blakeley et al., 1982; Cunnane and Stjärne, 1984). It has been suggested that the different latencies may be due to the distance between the release site and the recording site, with long latencies, for example, arising from more distant release (Blakeley et al., 1984). However, this hypothesis is not supported by the observation that there is no correlation between distance (between recording site and the coincident NCT) and the latency of the DE (Supplementary Fig. 2B). The basis of the variable latencies of DEs is more likely to be differing conduction velocities of axons innervating an SMC.

### Correlations in the amplitudes of NCTs and the first derivative of the EJP

The significant effect of distance, between the recording site and the site of NCT occurrence, and the amplitude of the first derivative of the EJP was first removed before the relationship between NCT amplitude and the amplitude of the first derivative of the EJP was investigated. There was no correlation between NCT amplitude and the resulting unstandardized residual of distance and the amplitude of the first derivative of the EJP. This finding represents a difference between sEJPs, which are positively correlated in amplitude with NCTs (Young et al., 2007), and the amplitude of the first derivative of the EJP, which are not correlated (Fig. 8; present study). This absence of a correlation between NCT amplitude and the amplitude of the first derivative of the EJP is surprising, but might arise because some DEs arise from the synchronous (evoked) release of neurotransmitter at different junctions on the same cell. For example, in a Poisson process with a mean probability of 0.99, the probability of having more two or more events on a given trial (using the condition that at least one event occurs; i.e. that an NCT is present) is 41%. Such confounding simultaneous release (at a distance) is not expected with spontaneous neurotransmitter release.

### Temporal correlation between NCTs and EJPs

NCTs correlate in timing with EJPs (Fig. 2B) but the latency between the peak EJP and the peak NCT is both greater than one frame and is very variable (Fig. 2A). Delays were greater than those reported between the sEJP and corresponding NCT (−30 to −57 ms; Young et al., 2007). The difference in delays between EJPs and NCTs (this study), and sEJPs and NCTs (Young et al., 2007) can be explained by the stimulation protocol used in this study. Electrical stimuli were provided at 1.5 Hz; this was at the beginning of every tenth confocal (acquisition) frame. This experimental design has the shortcoming that the first sign of an NCT (e.g. Fig. 1A, frame ii)—a spatially small (‘very focal’) rise in fluorescence—is of insufficient amplitude to be detected by the image analysis algorithm, which detects events based on both their amplitude, with respect to the noise, and area (Brain et al., 2003). Detection of the NCT took place only when the fluorescence had spread slightly (e.g. Fig. 1A, frame iii). The result is that temporal measurements have an error of at least one frame.

## Conclusions

1. DEs coincide with NCTs in the impaled cell but not in nearby cells. DEs thus reflect the action of packets of ATP on the impaled SMC. SMCs are poorly coupled electrically. 2. The relationship between DE amplitude and distance suggests that the junctional current is low close to the electrode, while the SMC is isopotential. 3. There is no correlation between DE and NCT amplitudes; the usefulness of NCTs lies primarily in locating the sites of impact of released ATP packets. But DEs may more reliably reflect the magnitude of packeted P2X-receptor-mediated response to released ATP packets. Both may give similar estimates of the release probability in individual sympathetic nerve varicosities.
