# Supplementary material for: Electrical and optical study of nerve impulse-evoked ATP-induced, P2X-receptor-mediated sympathetic neurotransmission at single smooth muscle cells in mouse isolated vas deferens
Source: Neuroscience. 2007 Aug 10;148(1):82–91. doi: 10.1016/j.neuroscience.2007.05.044 (PMC2151008; doi:10.1016/j.neuroscience.2007.05.044)
Supplement: Supplementary material [file mmc1.doc]

**Supplementary material: Modelling K^+^ flux**

Consider the one-dimensional diffusion of K^+^ from a fixed source, such as an electode tip within a long thin smooth muscle cell, with first-order removal of K^+^ along the length of the muscle cell. K^+^ diffuses away from this fixed source (at x=0) that is held at a constant concentration C(x=0, t)=A. If K^+^ is removed by a first-order process (with rate constant k), then for each point at distance x and time t,

In the steady state:

so:

This is a homogenous linear second-order differential equation with characteristic roots,

$r_{1}=\sqrt{\frac{k}{D}}$ and

with a general solution (Kreyszig, 1993),

In this case, however, C(x) goes to 0 as x goes to infinity, so c_1_=0. Substituting C=A at x=0 gives c_2_=A, so:

This implies that the concentration falls exponentially away from the source with a space constant of $r_{1}=\sqrt{\frac{k}{D}}$.

To calculate k, consider that the ion flux across a membrane is given by the Goldman, Hodgkin, Katz constant field equation (Goldman, 1943; Hodgkin & Katz, 1949):

so

Note that dj/d[K]_i_ is independent of [K]_i_ (as long as the membrane potential is held constant) and so the change in flux is proportional to the change in ion concentration. So for a change in K^+^ concentration above the resting concentration (Δ[K]), the flux is

To calculate the flux at a given point, segment length ’dl’, radius of cylindrical cell ’r‘, we have:

dω_K_=P_K_π2r.dl

So

Now, the concentration is given by

So

But is the (inward) flux, so

Substituting dJ_K_ from above gives:

or

where

So, the space constant for the excess K^+^ is:

Substituting the values from Table 1 gives a space constant of 585 µm.

**Table 1**

| **Parameter** | **Value** | **Reference** |
| --- | --- | --- |
| Membrane K^+^ permeability (P_K_) | 7.17x10^-8^ cm.s^-1^ | Brading (1971) |
| Faraday’s constant (F) | 96,485 J·V^-1^·mol^-1^ |  |
| Membrane Potential (ϕ_m_) | 0.075 V | This study. |
| Idea Gas Constant (R) | 8.314 J·K^-1^·mol^-1^ |  |
| Temperature (T) | 306 K | Used in study. |
| K^+^ diffusion coefficient | 3.7 X 10^2^ µm^2^.s^-1^ | For ventricular muscle (Cleeman & Gaughan, 1984) |
| Cell radius (r) | 0.0004 cm | This study. |
