# Supplementary material for: Electrical and optical study of nerve impulse-evoked ATP-induced, P2X-receptor-mediated sympathetic neurotransmission at single smooth muscle cells in mouse isolated vas deferens
Source: Neuroscience. 2007 Aug 10;148(1):82–91. doi: 10.1016/j.neuroscience.2007.05.044 (PMC2151008; doi:10.1016/j.neuroscience.2007.05.044)
Supplement: Supplementary Figure 1 [file mmc2.pdf]

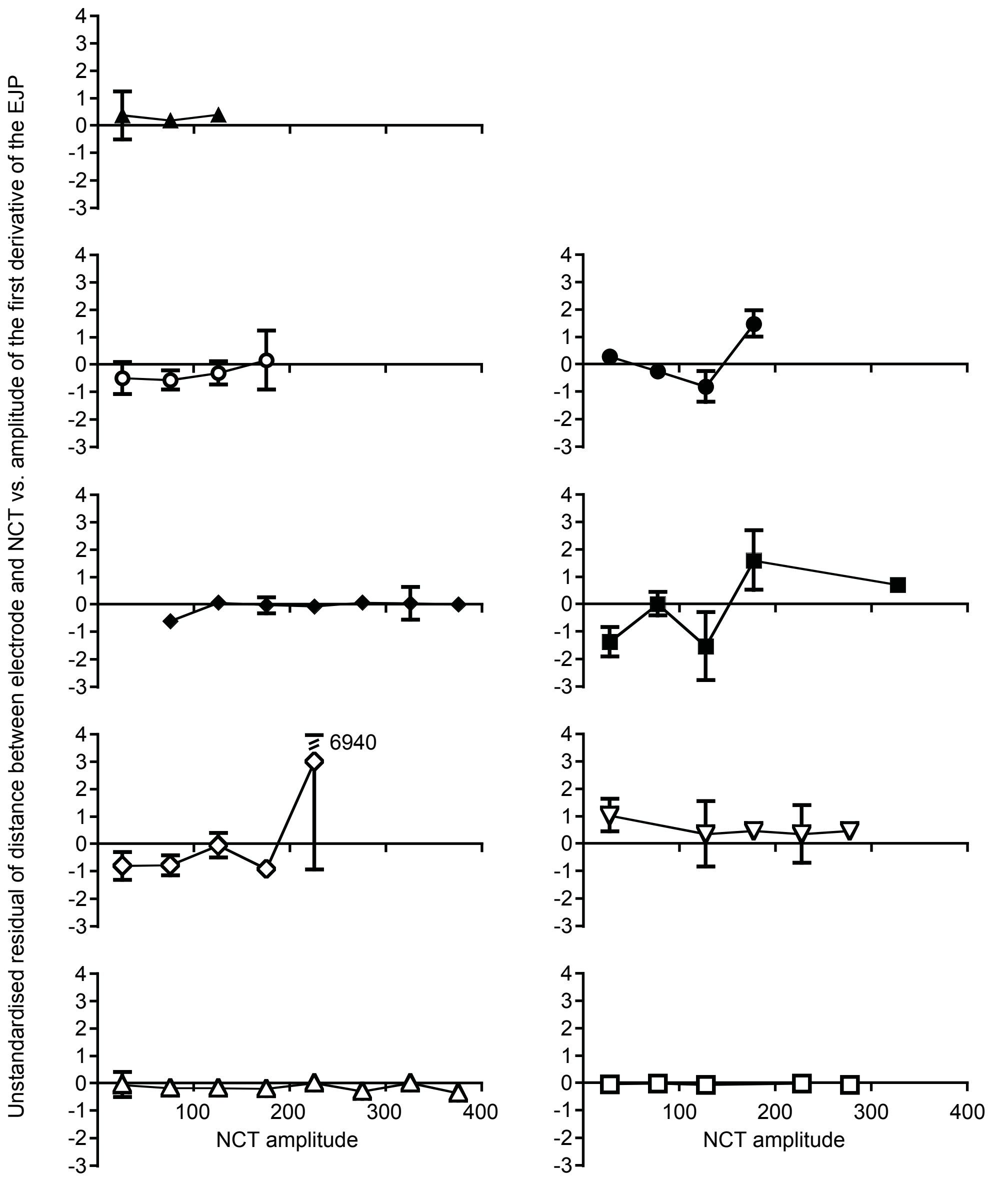

Supplementary Figure 1. There is no correlation between the NCT amplitude and the unstandardised residual of the distance between the electrode and the NCT vs. the amplitude of the first derivative of the EJP (Spearman two-tailed correlation performed on each data set; *r* ranged from -0.5 to +0.8, *P* = NS for all). Individual plots represent the relationships for each preparation. Unstandardised residuals are binned for presentation; data are mean ± SEM. Symbols correspond to those used in Fig. 2.
