# Supplementary material for: Electrical and optical study of nerve impulse-evoked ATP-induced, P2X-receptor-mediated sympathetic neurotransmission at single smooth muscle cells in mouse isolated vas deferens
Source: Neuroscience. 2007 Aug 10;148(1):82–91. doi: 10.1016/j.neuroscience.2007.05.044 (PMC2151008; doi:10.1016/j.neuroscience.2007.05.044)
Supplement: Supplementary Figure 2 [file mmc3.pdf]

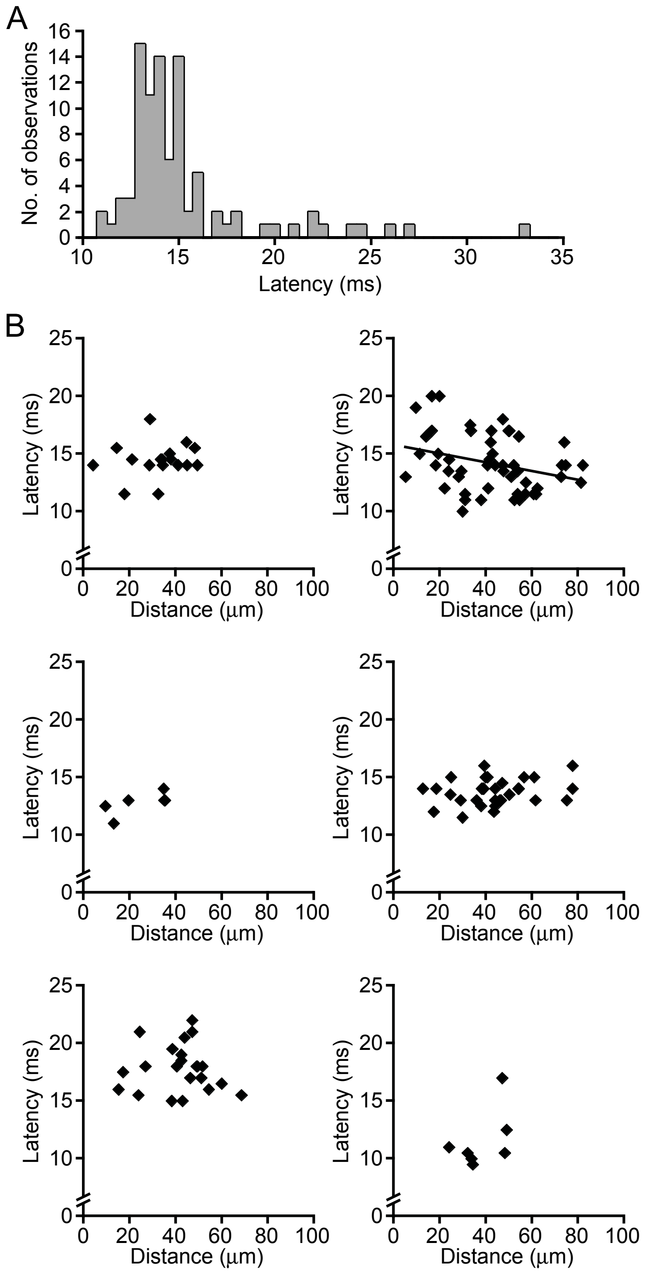

Supplementary Figure 2. There was no correlation between distance (between site of NCT and the recording site) and the latency of the corresponding DE. *A*, DEs occur with variable latency with respect to the stimulus. *B*, In five out of six preparations, there was no correlation between distance and the latency of the corresponding DE (Linear regression, $F = 0.1 - 3.6$, $r^2 = 0 - 0.48$, $P = 0.13 - 0.83$). In one of six preparations, there is a negative relationship between distance and DE latency (Linear regression, $F = 5.0$, $r^2 = 0.09$, $P < 0.05$). Data are presented for the six preparations (of a total of nine) where there were sufficient occurrences of DEs to perform this analysis.
